# Psychological Distress and Happiness of Men Who Have Sex With Men and Transgender People During the Coronavirus Disease-19 Pandemic: Is There a Need for Public Health Policy Intervention?

**DOI:** 10.3389/fpubh.2021.647548

**Published:** 2021-09-14

**Authors:** Ni Wayan Septarini, Jacqueline Hendriks, Bruce Maycock, Sharyn Burns

**Affiliations:** ^1^Department of Community and Preventive Medicine, Faculty of Medicine, Udayana University, Denpasar, Indonesia; ^2^Curtin School of Population Health, Curtin University, Perth, WA, Australia; ^3^Collaboration for Evidence, Research and Impact in Public Health, Curtin University, Perth, WA, Australia; ^4^European Centre for Environmental and Human Health, College of Medicine and Health, University of Exeter, Exeter, United Kingdom

**Keywords:** mental health, happiness, COVID-19, MSM, transgender, intervention, psychological distress

## Abstract

Since the global onset of COVID-19 in early 2020, the disease has significantly impacted mental health. This impact is likely to be further exacerbated for groups who were already marginalized. This paper shares results from a broader study of men who have sex with men (MSM) and transgender people in Bali, Indonesia and includes a focus on psychological distress and happiness during the COVID-19 pandemic; applying sociodemographic and epidemiological characteristics as potential mediators. Psychological distress and the level of happiness were measured by The Kessler Psychological Distress (K10) and the Subjective Happiness Scale (SHS). A cross-sectional survey was conducted from July to September 2020. Of the 416 participants, complete data were available for 363 participants. The majority of participants were aged 26–40 years, currently single, were born outside Bali, were currently living in an urban area, and over one-third were living with HIV. While all were MSM, the majority identified as homosexual/tend to be homosexual (71.3%), however 54 (14.9%) identified themselves as heterosexual. The majority (251, 69.1%) reported moderate to very high psychological distress during the COVID-19 pandemic. The binary logistic regression analysis identified five factors to be significantly associated with higher psychological distress: being a student, reporting higher levels of stigma, had ever experienced discrimination, felt better prior to the COVID-19 pandemic, and less happy than the average person. When homosexual were compared with heterosexual participants, those who identified themselves as being homosexual reported significantly lower psychological distress compared to those identified themselves as heterosexual, which may be associated with these participants not disclosing their status as MSM and the stigma around MSM. Those who considered themselves to be less happy than the average person (316, 87.1%) were more likely to live with a partner and to report moderate to very high psychological distress. Based on the findings, interventions should focus on strategies to reduce stigma, provide non-discriminatory services, and improve access to essential health services.

## Introduction

The first COVID-19 case in Bali was identified amongst an international tourist in February 2020, with community transmission increasing from June 2020 onwards (1). In December 2020, the cumulative cases were at 16,947 with a case fatality rate of 2.95% (around 500 deaths) (2). As per 15th of August 2021, the cumulative cases were 96,027 of which 85.28% (81,892) are recovered and 2,709 (2.82%) deaths (3). Throughout this pandemic, the government; related stakeholders, individuals organizations, and businesses throughout Indonesia have implemented control measures to reduce transmission of COVID-19. The control measures include stay-at-home orders, physical distancing, wearing face masks, and regular hand washing (4). In July 2020, the Bali Government announced a plan to resume all activities in the island *via* a “New Era of Life Order Protocol” that involved a strategy of reopening across three stages (1). However, due to the increase number of COVID-19 cases since July 2021, the Government of Indonesia has introduced level 4 of COVID-19 restriction known as emergency PPKM (Pemberlakuan Pembatasan Kegiatan Masyarakat) in order to restrict public activities and to reduce the COVID-19 transmission (5). The COVID-19 pandemic has affected mental well-being worldwide, even though individual mechanisms for coping may differ. Researchers have investigated transmission pathways for COVID-19, treatment options, impacts upon physical, and mental health amongst the general population, patient groups, and health providers (6). Evidence regarding the effects of the pandemic on mental health of marginalized groups remains limited, especially for MSM and transgender, who exist as hidden communities in many societies. In Indonesia, MSM and transgender women (known in Bali as “waria”) report difficulties associated with social stigma, violence, persecution, and other legal challenges which restrict the development of inclusive public policy (7) that leads them to become ‘hidden populations’ who are difficult to reach (8). Although MSM and waria are “accepted” in some parts of Indonesia, they continue to experience rejection due to family, cultural, and religious reasons in many regions of the country.

Several studies have been conducted to explore the well-being of MSM and transgender people globally during the COVID-19 pandemic. A study conducted in Brazil in 2020 found that 7.9% of MSM and transgender participants to report low psychological well-being (9), whereas research conducted in Mexico suggested high levels of depression among MSM and transgender women during the COVID-19 pandemic when compared to before the pandemic (10). A systematic review from available evidence revealed that overall MSM and transgender individuals suffered from disproportionate negative influence of stressors linked to the pandemic due to pre-existing vulnerabilities (11). The findings also suggested MSM and transgender peoples vulnerability were increased by mental health, economic deficit, and physical vulnerability during the pandemic (11). To the best of our knowledge, there was no literature available specifically focusing on happiness amongst MSM and transgender people during the COVID-19 pandemic. To date there have been a few published studies exploring the effect of COVID-19 on mental health in Indonesia, however these focus on the general population. A study of Indonesian adults (*n* = 8,000+) found levels of anxiety were highest among younger people and females. A study of healthcare workers (*n* = 227) found more than one third of respondents reported high levels of anxiety which was attributed to lower resilience. During non-pandemic times, MSM and transgender individuals in Indonesia have been found to be more likely to experience mental health issues compared to the general population (12). For example, a study in Bali in 2015 reported a high level of social anxiety and depression amongst MSM (13). To the best of our knowledge there have been no published studies focusing on psychological distress and happiness conducted among these communities in Bali during the current pandemic.

This paper examines the psychological distress and happiness of MSM and transgender people in Bali during the COVID-19 pandemic. Factors related to psychological distress and happiness amongst MSM and transgender people in Bali during the COVID-19 pandemic were measured, and comparisons made to participants' self-perception of these measures prior to the pandemic.

## Methodology

### Study Design, Participants, and Procedure

Data presented in this paper is part of a broader community-engaged research study exploring attitudes, behaviors and experiences of the MSM and transgender communities in Bali, Indonesia (14). In the third phase of this study, a cross-sectional survey was hosted on the Qualtrics platform from 6th of July to 28th of September 2020. Most participants completed the online survey independently; however research partners read the survey aloud for those with low literacy levels. Given the estimation of the MSM population in Bali, of around 14,000 adults, to obtain 95% confidence level and 5% precision (margin of error), the required sample for the survey was calculated to be 374 respondents (15). The detail of survey methodology can be found in the protocol paper of the project (14).

A convenience sample of participants were recruited, assisted by 10 Balinese-based research partners who were staff of various non-government organizations (NGOs) focusing on the health of marginalized communities including MSM and transgender people. Partner-driven sampling technique was used to recruit participants. As part of this community-engaged research the research partners have been involved in each stage of the research. These partners recruited participants purposively and *via* snowballing technique. Social media was also used to recruit participants. Each research partner recruited at least 40 participants within four rounds of data collection. Interested participants were provided the survey link *via* email or WhatsApp. Eligible participants were aged 18 years or older, Indonesian citizens who had lived in Bali for at least 6 months and intended to remain there for at least the next 6 months, identified as male or transgender (waria), and had engaged in sexual activity with a man or transgender person in the last 6 months.

All survey responses were anonymous and data stored on a secure University network. Broadly, the survey captured various attitudes, behaviors, and experiences of MSM and transgender, however this paper focuses on data relating to: sociodemographic characteristics, the Kessler Psychological Distress (K-10) scale (16), and the Subjective Happiness Scale (SHS) (17). The full survey is available on request to the corresponding author.

### Measures

The structured online survey included previously validated questions and scales (14, 18–21). It was originally written in English and then translated into Bahasa Indonesia. Face and content validity testing was conducted with the research partners and Indonesian public health experts.

Demographic characteristics including age, gender, sexual identity, marital status, education level, daily activities, place of birth, and residential district were collected. Other items included family attitude toward MSM/transgender status, social networking before and during the COVID-19 pandemic, number of regular partner(s), stigma [using the 12-item short version of the HIV stigma scale (21)], discrimination [based on previously validated measures (22)], and HIV status.

### Psychological Distress Assessment

The K10 scale was used to measure psychological distress (16). The ten-item scale is used widely for epidemiological and clinical purposes as a simple self-report tool to identify persons who require further assessment for depression and anxiety (16). Scores range from 1 to 50 and were collapsed into four categories: low (10–15); moderate (16–21); high (22–29); and very high (30–50).

As this survey was administered during the COVID-19 pandemic, participants were also asked to reflect how they were feeling, in terms of psychological distress, prior to the pandemic (1 year ago). Three responses were provided: (1) the same; (2) previously my feeling/condition was better compared to now; and (3) previously my feeling/condition was worse compared to now.

### Happiness Assessment

The Subjective Happiness Scale (SHS), is a four-item self-report scale used to assess a person's overall happiness (7-point Likert scale) (17). The first two items ask participants to categorize themselves using an absolute rating as being a happy person and a happiness rating relative to their peers. The two last items present short phrases describing happy or unhappy people and ask respondents to identify the degree to which these scenarios best describe them (17). An overall score is calculated by averaging the answers. Scores range from 1 to 7, with higher scores reflecting greatest happiness (17). This scale has been used and validated in 14 different studies with over 2,700 participants (17).

### Data Analysis

Data were analyzed using SPSS v.26. Descriptive analyses were used to describe the research variables. Mean, standard deviation, and range were calculated for continuous variables (age) and for each scale (K10 and SHS). For inferential analyses, K10 was categorized as low psychological distress (score 10–15) and moderate to very high (score 16–50) (23). SHS was categorized as less happy than average person (score <5.6) and happier than average person (score ≥ 5.6) (17). Initially, variables associated with psychological distress were identified by comparing the two categories on the K10 scale and the two categories on the SHS scale using a chi-square test. Statistical significance was set at *p* < 0.05. Subsequently, binary logistic regression reported the strength of association, which generated odds ratio (OR) and 95% confident interval (CI). Multivariate analyses provided adjusted OR (AOR), with 95% CI, by adjusting for demographics (gender, age, sexual identity, education level, daily activity, place of birth, and residential district) and epidemiological characteristics (family attitude toward MSM/transgender status, social networking before and after COVID-19 pandemic, number of regular partners, stigma, discrimination, and overall feeling/condition before COVID-19 pandemic).

### Ethical Approval

The study was approved by the Human Ethics Committee, Faculty of Medicine, Udayana University/Sanglah Hospital, Bali, Indonesia (No: 2521/UN14.2.2.VII.14/LP/2019) and the Curtin University Human Research Ethics Committee, Australia (HRE 2019-0759). This research was carried out in accordance with the Declaration of Helsinki with written informed consent obtained from all participants.

## Results

Responses were received from 416 MSM and transgender individuals living in Bali, Indonesia and complete data were available for 363 participants. The mean age (SD) of participants was 32.46 (7.83) years and 68.3% were aged 26–40 years. The majority identified as male (72.5%) and indicated their sexual identity to be homosexual/tend to be homosexual (71.4%). More than half of the participants had completed senior high school (52.3%) and over half were working full time (55.6%). Most participants were born outside Bali Province (58.9%) and lived in urban areas (85.7%). Nearly 30% of participants reported they were either married or living with a partner. Just over a half of participants (56.5%) had not disclosed their identity as either MSM or transgender to their family members. Around 35% were living with HIV (Table 1). Individual items for psychological distress and happiness are described in Tables 2, **4**.

**Table 1:** Characteristics of the study participants.

**Characteristics**	**Total**, ***n*** (**%)**
**Total participants**	**363**
**Age (in years)**	
Mean (±SD)	32.46 (7.83)
Range	41
**Age groups**	
18–25	63 (17.4)
26–40	248 (68.3)
41–60	52 (14.3)
**Gender**	
Male	263 (72.5)
Transgender/waria	100 (27.5)
**Sexual identity**	
Heterosexual/tend to be heterosexual	54 (14.9)
Homosexual/tend to be homosexual	259 (71.3)
Bisexual	50 (13.8)
**Education level**	
No or elementary school	39 (10.7)
Junior high school	61 (16.8)
Senior high school	190 (52.3)
Diploma or higher	73 (20.1)
**Marital status**	
Single (not married, widow)	262 (72.2)
Married	30 (8.3)
Living with a partner	71 (19.6)
**Daily activities**	
Regular/full time job	2,020 (55.6)
School/college	46 (12.7)
No job/no school	58 (16)
Home duties/others	57 (15.7)
**Place of born**	
Bali	149 (41)
Java	133 (36.6)
Others	81 (22.3)
**Current living area**	
Urban	311 (85.7)
Rural	52 (14.3)
**HIV+ status**	
Yes	130 (35.8)
No	218 (60.1)
Do not know/have never tested for HIV	15 (4.1)
**Family attitudes**	
All accept	97 (26.7)
All/some reject	61 (16.8)
Do not know about MSM status	205 (56.5)

**Table 2:** Level of psychological distress among the study participants (*n* = 363).

**Anxiety and depression checklist (K10) (last 4 weeks)**					
**About how often did you feel tired out for no good reason?**					
None	134 (36.9)	Most of the time	7 (1.9)		
A little of the time	176 (48.5)	All the time	0 (0.0)		
Some of the time	46 (12.7)				
**About how often did you feel nervous?**					
None	149 (41.0)	Most of the time	7 (1.9)		
A little of the time	150 (41.3)	All the time	2 (0.6)		
Some of the time	55 (15.2)				
**About how often did you feel so nervous that nothing could calm you down?**					
None	180 (49.6)	Most of the time	4 (1.1)		
A little of the time	139 (38.3)	All the time	0 (0.0)		
Some of the time	40 (11.0)				
**About how often did you feel hopeless?**					
None	141 (38.8)	Most of the time	6 (1.7)		
A little of the time	165 (45.5)	All the time	2 (0.6)		
Some of the time	49 (13.5)				
**About how often did you feel restless or fidgety?**					
None	100 (27.5)	Most of the time	8 (2.2)		
A little of the time	186 (51.2)	All the time	1 (0.3)		
Some of the time	68 (18.7)				
**About how often did you feel so restless you could not sit still?**					
None	142 (39.1)	Most of the time	5 (1.4)		
A little of the time	156 (43.0)	All the time	1 (0.3)		
Some of the time	59 (16.3)				
**About how often did you feel so depressed?**					
None	169 (46.6)	Most of the time	6 (1.7)		
A little of the time	142 (39.1)	All the time	1 (0.3)		
Some of the time	45 (12.4)				
**About how often did you feel that everything was an effort?**					
None	66 (18.2)	Most of the time	28 (7.7)		
A little of the time	104 (28.7)	All the time	47 (12.9)		
Some of the time	118 (32.5)				
**About how often did you feel so sad that nothing could cheer you up?**					
None	124 (34.2)	Most of the time	9 (2.5)		
A little of the time	166 (45.7)	All the time	6 (1.7)		
Some of the time	58 (16.0)				
**About how often did you feel worthless?**					
None	187 (51.5)	Most of the time	7 (1.9)		
A little of the time	131 (36.1)	All the time	0 (0.0)		
Some of the time	38 (10.5)				
**How do you feel/the condition that you conveyed before the COVID-19 pandemic (1 year ago)**					
The same	113 (31.1)	Previously my feeling/condition was better	226 (62.3)	Previously my feeling/condition was worst	24 (6.6)
**K10 score (total)**					
Mean (SD)	18.72 (5.7)				
Range (minimum–maximum)	30 (10–40)				
**Level of psychological distress (K10 categories)**					
Low (score 10–15)	112 (30.9)	High (score 22–29)	81 (22.3)		
Moderate (score 16–21)	156 (43.0)	Very high (score 30–50)	14 (3.9)		

### Psychological Distress

The mean (SD) K10 score was 18.72 (5.7); with scores ranging from 10 to 40. Based on the four categories of psychological distress, participants were most likely to report moderate psychological distress (156, 43.0%). Only 3.9% (*n* = 14) participants reported very high psychological distress while 30.9% (*n* = 112) reported low levels of psychological distress (Table 2). After collapsing psychological distress into two categories, 69.1% (*n* = 251) of participants reported moderate to very high psychological distress.

Univariate analyses revealed eight variables (sexual identity, primary daily activity, family attitude about the sexual identity, number of regular partner, experience of stigma, experience of discrimination, overall feeling before the COVID-19 pandemic, and level of happiness) to be significantly associated with psychological distress (Table 3).

**Table 3:** Factor associated with psychological distress among the study population (based on K10 score).

**Characteristics/variables**	**Low (score 10–15), *n* (%)**	**Moderate to very high (score 16–50), *n* (%)**	**Univariate analyses**			**Multivariate analyses**		
			***P***	**OR**	**95%Cls**	***P***	**AOR**	**95% Cls**
Total study participants (*n* = 363)	112 (30.9)	251 (69.1)						
**Gender**								
Male	87 (33.1)	176 (66.9)	1			1		
Transgender/waria	25 (25)	75 (75)	0.138	1.483	0.881–2.495	0.435	0.725	0.324–1.623
**Age**								
18–25	19 (30.2)	44 (69.8)	1			1		
26–40	74 (29.8)	174 (70.2)	0.960	1.015	0.556–1.855	0.872	1.064	0.500–2.266
41–60	19 (36.5)	33 (63.5)	0.470	0.750	0.344–1.636	0.962	0.976	0.360–2.650
**Sexual identity**								
Heterosexual	7 (13.0)	47 (87.0)	1			1		
Homosexual	85 (32.8)	174 (67.2)	0.005	0.305	0.132–0.703	0.046	0.409	0.170–0.984
Bisexual	20 (40.0)	30 (60.0)	0.003	0.223	0.084–0.592	0.052	0.355	0.125–1.007
**Education level**								
No/Elementary school	9 (23.1)	30 (76.9)	1			1		
Junior high school	13 (21.3)	48 (78.7)	0.835	1.108	0.422–2.906	0.845	1.119	0.363–3.443
Senior high school	66 (34.7)	124 (65.3)	0.161	0.564	0.253–1.258	0.340	0.613	0.224–1.675
Diploma/University	24 (32.9)	49 (67.1)	0.281	0.613	0.251–1.493	0.686	0.795	0.262–2.415
**Daily activity**								
Regular/full time job	72 (35.6)	130 (64.4)	1			1		
School/college	6 (13.0)	40 (87.0)	0.005	3.692	1.494–9.128	0.005	4.009	1.530–10.503
No job/no school	16 (27.6)	42 (72.4)	0.255	1.454	0.764–2.768	0.357	1.383	0.693–2.759
Home duties/others	18 (31.6)	39 (68.4)	0.570	1.200	0.640–2.249	0.668	1.163	0.584–2.317
**Marital status**								
Single (not married, widow)	81 (30.9)	181 (69.1)	1			1		
Married	12 (40.0)	18 (60.0)	0.314	0.671	0.309–1.459	0.117	0.485	0.196–1.199
Living with a partner	19 (26.8)	52 (73.2)	0.498	1.225	0.681–2.203	0.316	0.709	0.362–1.389
**Place of birth**								
Bali	53 (35.6)	96 (64.4)	1			1		
Java	39 (29.3)	94 (70.7)	0.265	1.331	0.806–2.198	0.577	1.201	0.632–2.281
Others	20 (24.7)	61 (75.3)	0.092	1.684	0.918–3.087	0.345	1.209	0.691–2.873
**Family attitude about sexual identify**								
All accept	30 (30.9)	67 (69.1)	1			1		
All/some reject	10 (16.4)	51 (83.6)	0.044	2.284	1.023–5.098	0.246	1.681	0.699–4.047
Family does not know the sexual identity	72 (35.1)	133 (64.9)	0.472	0.827	0.493–1.388	0.399	0.766	0.412–1.423
**Residential district**								
Urban	92 (29.6)	219 (70.4)	1			1		
Rural	20 (38.5)	32 (61.5)	0.201	0.672	0.365–1.236	0.958	1.022	0.459–2.272
**Networking with MSM friends before COVID-19 pandemic**								
Yes	60 (29.1)	146 (70.9)	1			1		
No	52 (33.1)	105 (66.9)	0.415	0.830	0.530–1.299	0.464	0.814	0.469–1.412
**Networking with MSM friends after COVID-19 pandemic**								
Yes	101 (31.6)	219 (68.4)	1			1		
No	11 (25.6)	32 (74.4)	0.427	1.404	0.745	0.865	1.080	0.443–2.636
**Number of regular partner**								
Does not have any regular partner	34 (34.7)	64 (65.3)	1			1		
1	62 (33.2)	125 (66.8)	0.794	1.071	0.640–1.793	0.945	1.024	0.524–2.001
>1	16 (20.5)	62 (79.5)	0.04	2.059	1.033–4.101	0.345	1.479	0.446–1.406
**Stigma**								
Lower stigma (score ≤ median)	69 (35.6)	125 (64.4)	1			1		
Higher stigma (score > median)	43 (25.4)	126 (74.6)	0.038	1.617	1.027–2.547	0.014	1.901	1.140–3.170
**Discrimination**								
Never experienced discrimination	34 (20.9)	122 (61.0)	1			1		
Ever experienced discrimination	78 (39.0)	129 (79.1)	0.000	2.426	1.512–3.892	0.001	2.464	1.464–4.147
**Overall feeling/condition before COVID-19 pandemic**								
The same/does not know	46 (40.7)	67 (59.3)	1			1		
Felt better	58 (25.7)	168 (74.3)	0.023	1.699	1.074–2.688	0.002	2.404	1.388–4.161
Felt worse	8 (33.3)	16 (66.7)	0.678	1.215	0.484–3.053	0.293	1.753	0.616–4.988
**Level of happiness**								
Happier than average person	28 (59.6)	19 (40.4)	1			1		
Less happy than average person	84 (26.6)	232 (73.4)	0.000	4.07	2.160–7.671	0.000	3.962	1.980–7.927
**HIV status**								
HIV+	35 (26.9)	95 (73.1)	1			1		
HIV–	73 (33.5)	145 (66.5)	0.983	0.295	0.295–3.304	0.078	0.602	0.342–1.059
Have never tested for HIV	4 (26.7)	11 (73.3)	0.588	0.722	0.222–2.347	0.664	0.736	0.184–2.937

Multivariate analyses found students (as the primary daily activity) were four times (AOR = 4.009, 95% CI: 1.530–10.503, and *p* = 0.005) more likely to report moderate to very high psychological distress compared to participants working full-time. Reporting higher (AOR = 1.901, 95% CI: 1.140–3.170, and *p* = 0.014) compared to lower stigma; ever having experienced discrimination (AOR = 2.464, 95% CI: 1.464–4.147, and *p* = 0.001) compared to never; feeling better before COVID-19 pandemic (AOR = 2.404, 95% CI: 1.388–4.161, and *p* = 0.002) compared to feeling the same; and self-identifying as less happy than the average person (AOR = 3.962, 95% CI: 1.980–7.927, and *p* = 0.000) were all significantly associated with higher psychological distress. Conversely, identifying as homosexual (AOR = 0.409, 95% CI: 0.170–0.984, and *p* = 0.046) was significantly associated with lower psychological distress compared to participants who identified themselves as heterosexual/tend to be heterosexual (Table 3).

### Level of Happiness

The mean (SD) SHS score was 4.74 (0.88), with score range 4.5 (Table 4). Based on two categories, most participants (316, 87.1%) self-reported to be less happy than the average person with, only 12.9% (*n* = 47) of participants considering themselves to be happier than the average person. None of the participants who identified themselves as heterosexual felt they were happier than the average person.

**Table 4:** Level of happiness among the study participants (*n* = 363).

**Subjective Happiness Scale (SHS) individual items**	**Mean (SD)**	**Range**
In general, I feel myself	5.17 (1.39)	6
When compared to most of my friends, I feel that I	5.11 (1.49)	6
Some people are generally very happy. They enjoy life no matter what happens, get the most out of everything. To what extent do these categories suit you?	4.98 (1.37)	6
Some people are generally not very happy. Even though they are not depressed/sad, they don't look as happy as they should. To what extent do these categories suit you?	4.31 (1.63)	6
SHS total (reverse the 4th question)	18.96 (3.53)	18
SHS score (reverse the 4th question)	4.74 (0.88)	4.5
**Level of happiness (** ***n*** **= 363)**	***n*** **(%)**	
Less happy than average person	316 (87.1)	
Happier than average person	47 (12.9)	

Univariate analyses found being a student, living with a partner, having more than one regular partner, and having moderate to very high psychological distress were significantly more likely to be associated with reporting to be less happy than the average person (Table 5). Multivariate analyses found only two associations to remain significant. Those living with a partner (AOR = 15.610, 95% CI: 2.074–117.471, and *p* = 0.008) and participants with moderate to high levels of psychological distress (AOR = 4.155, 95% CI: 2.150–8.032, and *p* = 0.000) were more likely to rate themselves as less happy than the average person (Table 5). Interestingly, in the multivariate analysis, a significant association was also found between happiness and those who felt better before the COVID-19 pandemic (AOR = 0.402, 95%CI: 0.180–0.898, and *p* = 0.026). This result suggests that after considering other variables, those who felt better before the pandemic were 2.5 times more likely to rate themselves as happier than average person compared to those who felt the same/did not know their feeling before the COVID-19 pandemic.

**Table 5:** Factor associated with level of happiness among the study population (based on SHS score).

**Characteristics /variables**	**Happier than average person**	**Less happy than average person**	**Univariate analyses**			**Multivariate analyses**		
			***P***	**OR**	**95%Cls**	***P***	**AOR**	**95% Cls**
Total study participants (*n* = 363)	47 (12.9)	316 (87.1)						
**Gender**								
Male	33 (12.5)	230 (87.5)	1			1		
Transgender/waria	14 (14.0)	86 (86.0)	0.713	0.881	0.450–1.727	0.251	1.969	0.619–6.265
**Age**								
18–25	7 (11.1)	56 (88.9)	1			1		
26–40	23 (13.3)	215 (86.7)	0.643	0.814	0.342–1.938	0.601	1.354	0.435–4.213
41–60	7 (13.5)	45 (86.5)	0.702	0.804	0.263–2.460	0.456	1.763	0.397–7.827
**Sexual identity**								
Heterosexual	0 (0.0)	54 (100.0)	1			1		
Homosexual	40 (15.4)	219 (84.6)	0.997	0.000	0.000	0.997	0.000	0.000
Bisexual	7 (14.0)	43 (86.0)	0.997	0.000	0.000	0.997	0.000	0.000
**Education level**								
No/Elementary school	7 (17.9)	32 (82.1)	1			1		
Junior high school	9 (14.8)	52 (85.2)	0.671	1.264	0.429–3.727	1.000	1.000	0.284–3.518
Senior high school	22 (11.6)	168 (88.4)	0.280	1.670	0.659–4.237	0.066	2.804	0.934–8.414
Diploma/University	9 (12.3)	64 (87.7)	0.421	1.556	0.531–4.558	0.400	1.723	0.486–6.106
**Daily activity**								
Regular/full time job	32 (15.8)	170 (84.2)	1			1		
School/college	1 (2.2)	45 (97.8)	0.038	8.471	1.127–63.681	0.232	3.715	0.432–31.965
No job/no school	7 (12.1)	51 (87.9)	0.480	1.371	0.571–3.292	0.754	0.846	0.297–2.412
Home duties/others	7 (12.3)	50 (87.7)	0.508	1.345	0.560–3.230	0.941	1.043	0.347–3.135
**Marital status**								
Single (not married, widow)	43 (16.4)	219 (83.6)	1			1		
Married	3 (10.0)	27 (90.0)	0.367	1.767	0.513–6.087	0.418	1.850	0.418–8.196
Living with a partner	1 (1.4)	70 (98.6)	0.010	13.744	1.859–101.639	0.005	19.463	2.474–153.124
**Place of birth**								
Bali	16 (10.7)	133 (89.3)	1			1		
Java	23 (17.3)	110 (82.7)	0.114	0.575	0.290–1.143	0.103	0.485	0.203–1.157
Others	8 (9.9)	73 (90.1)	0.838	1.098	0.448–2.688	0.699	0.816	0.291–2.289
**Family attitude about sexual identify**								
All accept	30 (30.9)	67 (69.1)	1			1		
All/some reject	10 (16.4)	51 (83.6)	0.142	2.212	0.766–6.388	0.378	1.767	0.498–6.269
Family does not know the sexual identity	72 (35.1)	133 (64.9)	0.373	1.360	0.692–2.673	0.156	2.139	0.748–6.113
**Residential district**								
Urban	42 (13.5)	269 (86.5)	1			1		
Rural	5 (9.6)	47 (90.4)	0.442	1.468	0.552–3.901	0.890	1.095	0.303–3.950
**Networking with MSM friends before COVID-19 pandemic**								
Yes	24 (11.7)	182 (88.3)	1			1		
No	23 (14.6)	134 (85.4)	0.400	0.768	0.416–1.419	0.445	0.716	0.304–1.687
**Networking with MSM friends after COVID-19 pandemic**								
Yes	45 (14.1)	275 (85.9)	1			1		
No	2 (4.7)	41 (95.3)	0.103	3.355	0.784–14.356	0.124	3.357	0.717–15.711
**Number of regular partner**								
Does not have any regular partner	18 (18.4)	80 (81.6)	1			1		
1	25 (13.4)	162 (86.6)	0.265	1.458	0.752–2.828	0.826	0.918	0.431–1.959
>1	5 (5.1)	74 (94.9)	0.013	4.162	1.347–12.867	0.071	3.034	0.911–10.103
**Stigma**								
Lower stigma (score ≤ median)	23 (11.9)	171 (88.1)	1			1		
Higher stigma (score > median)	24 (14.2)	145 (85.8)	0.507	0.813	0.440–1.500	0.234	0.649	0.318–1.324
**Discrimination**								
Never experienced discrimination	26 (13.0)	174 (87.1)	1			1		
Ever experienced discrimination	21 (12.9)	142 (87.1)	0.974	1.010	0.546–1.871	0.416	0.705	0.304–1.635
**Overall feeling/condition before COVID-19 pandemic**								
The same/does not know	12 (10.6)	101 (89.4)	1			1		
Felt better	31 (13.7)	195 (86.3)	0.420	0.747	0.368–1.518	0.026	0.402	0.180–0.898
Felt worse	4 (16.7)	20 (83.3)	0.406	0.594	0.174–2.031	0.238	0.444	0.115–1.712
**Psychological distress (K10)**								
Low	28 (25.0)	84 (75.0)	1			1		
Moderate to very high	19 (7.6)	232 (92.4)	0.000	4.070	2.160–7.671	0.000	4.525	2.210–9.265
**HIV status**								
HIV+	17 (13.1)	113 (86.9)	1			1		
HIV–	28 (12.8)	190 (87.2)	0.950	1.021	0.535–1.948	0.814	1.103	0.489–2.486
Have never tested for HIV	2 (13.3)	13 (86.7)	0.978	0.978	0.203–4.717	0.297	2.952	0.385–22.612

## Discussion

This cross-sectional study provides unique understandings of the impact the first 5–7 months of the COVID-19 pandemic has had on psychological distress and happiness amongst MSM and transgender people living in Bali, Indonesia. At the time of data collection no peer-review publications had reported findings describing psychological distress or happiness amongst the Balinese MSM or transgender community. A recent study amongst university students in Indonesia found 72% of reported mild depression (24) and a study within the general population reported people under 50 years experienced higher anxiety during the COVID-19 pandemic compared to older participants (25). Another study found 48% of Indonesian women experienced psychological distress as an impact of working from home (26). However, none of these studies employed the K10 to measure psychological distress.

Globally studies across various population groups have employed a range of measures to determine psychological distress (6, 23, 27–32). Studies outside Indonesia conducted during the COVID-19 pandemic have reported the levels of psychological distress using the K10 to be similar to this study (23, 27, 31, 32). An Australian study found 62.5% of adults reported moderate to very high psychological distress (23). Studies conducted in Jordan found nearly 70% of university students reported severe psychological distress (32), whereas, one third of University teachers (31.4%) in the same country reported severe distress levels (31). A study conducted in New Zealand during a COVID-19 lockdown found one-third of participants to report a K10 score above 12 (moderate to severe psychological distress) (27). The differences in psychological distress among people from different countries may be associated with different characteristics of participants and/or varying impact of the pandemic in terms of isolation measures and infection rates. For example, New Zealanders may have felt they were safer in their own country than elsewhere (27). Another study conducted in Italy also found more than half of participants to report no psychological distress (28). Furthermore, a study amongst medical students in Saudi Arabia found 44.5% of participants reported no distress during the COVID-19 pandemic while 12.8% reported severe distress (30).

Interestingly, in this study participants who identified as homosexual reported lower levels of psychological distress compared to their heterosexual identifying peers. This may be a result of social stigma around sexual orientation in Indonesian society. Those who identify as homosexual may have already embraced and accepted their sexual identity. The MSM participants who identified themselves as heterosexual, are likely to have not gone through this process. In Indonesia there is significant social stigma associated with identifying as homosexual or bisexual (12). These participants are likely to be struggling with their sexual identity and many may also be hiding their identity from family or friends or living a double life which may contribute to their higher levels of psychological distress.

This study also found MSM and transgender students were four times more likely to report moderate to very high psychological distress in comparison to peers currently working full-time. This phenomenon could be due to students experiencing higher levels of stress associated with studying and adapting to new ways of learning (for example, online learning). University students in France reported more than 60% of participants experienced moderate to severe life stress (33), however a study among medical school students in China found <4% reported “at least moderate” levels of anxiety during the COVID-19 pandemic (34). The lower prevalence of distress amongst working group participants may also reflect higher levels of well-being and resilience from having overcome past adversities and experiencing fewer daily disruptions which may help protect subjective happiness (27, 35). Higher levels of resilience has been suggested to reduce fear and anxiety due to COVID-19 (35). These findings warrant further investigation regarding the potential protective factors of employment during COVID-19 on the impact of psychological distress.

Other factors that significantly influenced psychological distress amongst MSM and transgender in this study were stigma and discrimination experiences. Participants who reported high levels of stigma or had ever experienced discrimination were more likely to report higher psychological distress. Stigma and discrimination are associated with poorer social and emotional health, consequently affecting levels of psychological distress (36). The impacts of the COVID-19 pandemic may act as additional stressors on stigma and discrimination. In countries that reported high levels of stigma toward sexual minority groups, lower life satisfaction were experienced by those who did not conceal their status in order to avoid discrimination (37). Furthermore, global evidence demonstrates that COVID-19 pandemic restrictions have been used as an excuse to discriminate, perpetuate stigma, and violence against LGBT individuals which may also increase distress levels (38). Moreover, LGBT populations, especially those who have other minority identities (such as ethnic minorities) face higher likelihood of unemployment, HIV, suicide and mental health problems, institutional discrimination and other human right violations compared to the general population (39). Analyses of three studies focusing on the health and happiness of LGBT individuals found minimizing discrimination to be positively associated with subjective well-being (40). A study of life satisfaction amongst sexual minority groups in 28 European countries revealed that life satisfaction varied greatly across countries, due to the structural stigma of those countries and was related to the varying demands that were required to conceal an individual's sexual orientation (37). These findings warrant further investigation regarding the role of factors related to stigma and discrimination on the mental health of LGBT communities during the COVID-19 pandemic.

Participants in this study who felt their overall feeling was better before the pandemic were 2.4 times more likely to report moderate to very high psychological distress compared to participants whose felt they had the same feeling before the pandemic. Similarly, longitudinal research in the United States (US) identified significant increases in distress during the emergence of the COVID-19 pandemic (41). However, the levels of distress were largely diminished in the weeks that followed, which might be associated with increased resilience (41). Furthermore, a national survey in Ireland revealed significant increases in symptoms of depression, stress, and anxiety upon entry into COVID-19 quarantine (42). Different levels of psychological distress across populations, including patients who experienced COVID-19 infection; individuals under quarantine; and the general population were reported in China (43). The prevalence of depression (29.2%) increased predominately in patients who experienced COVID-19 infection (43), while COVID-19 patients and the general public reported a greater proportion of severe depressive symptoms compared to those in quarantine (43).

The majority of MSM and transgender participants in this study viewed themselves as less happy than the average person, which may be due to fear of COVID-19. A study examining the relationship between hope, resilience, and subjective happiness in Turkey revealed that subjective happiness was mediated by a fear of COVID-19 (35). However, this study was unable to compare the subjective happiness level before and after COVID-19. Compared to 2016, the proportion of unhappiness in the general population in China doubled during the COVID-19 pandemic (44). To date, no peer-review publications appear to have reported happiness amongst MSM and transgender communities during the COVID-19 pandemic. However, a US study revealed that the majority of MSM participants had decreased quality of life and increased anxiety due to COVID-19 which was similar to the findings of this study (45).

Comparable to the psychological distress findings, when happiness was considered, none of the heterosexual participants reported to be happier than average person. Participants who were currently living with a partner were 19 times more likely to rate themselves as less happy than average person compared to those who were currently living alone. A nationally representative study in the US revealed that stress related to sexual minority status in earlier life may accumulate over time, resulting in lower happiness later in life (46). Moreover, those with current different-sex partners but histories of same or both-sex partners may be disadvantaged and the heterosexual identified group may also have faced pressure to act “closeted” and may be unhappy with their current sexual arrangements (47, 48). Furthermore, current and lifetime measures of the sex of sexual partners revealed important happiness differences, which advised that stability in sex of sexual partners was associated with better well-being/happiness (48).

Psychological distress and level of happiness were highly associated. In this study, those who reported moderate to very high psychological distress were four times more likely to be less happy compared to those who reported low psychological distress. Likewise, compared to happier people, those who were less happy were also four times more likely to report moderate to very high psychological distress. A study in Turkey found distress and happiness to have a negative correlation (inversely correlated) and positivity to be a potential mediator on COVID-19 perceived risk, death distress, and happiness (49). Individual's positive views about self, life and future (positivity) was positively associated with happiness and negatively associated with death distress (49). Therefore, it has been suggested positivity is an important aspect of developing strength-based programs aiming to lessen psychological distress and increase happiness (49).

### Study Strengths and Limitations

This study provides baseline findings about psychological distress and happiness amongst specific marginalized populations in Bali, Indonesia. The study achieved a sufficient sample during a crisis period (14). However, the study is subject to a number of limitations. Participants predominantly resided in urban areas (the capital city of Bali). Considering the restriction of movement and social distancing during the COVID-19 pandemic, findings might be more generalizable to Indonesian urban, compared to rural areas. The experiences of MSM and transgender people living in Bali, may differ in other areas in Indonesia. Findings of this study were limited to MSM and transgender who have accessed sexual health clinics or an NGO outreach service in Bali; hence, the study may not be generalizable to those who live in remote areas those not currently connected to a health service and/or those who may have more limited access due to COVID-19 restriction (50). The survey, which began development before the pandemic, asked limited questions specific to the COVID-19 pandemic. The study was unable to assess the effect of the COVID-19 pandemic on mental health and happiness of MSM and transgender in Bali since the baseline data were not available. Further research is warranted to provide a deeper understanding of the impact of COVID-19 on MSM and transgender people in Bali, Indonesia.

### Policy Implication and Future Research

This study provides an important insight into to the mental health and happiness of sexual minority groups which are sometimes neglected and highly at-risk (51). Mental health and consequently access to mental health services are stigmatized in some countries like Indonesia, and the COVID-19 pandemic makes access more difficult due to isolation measures. The findings of this study suggest that psychological distress amongst MSM and transgender people is a significant public health issue which is influenced by many factors. The psychological impact of COVID-19 may also exacerbate mental health burden and vulnerability among these already at risk communities (e.g., anxiety, depression, and suicidal thoughts) (52). Given stigma and discrimination have been found to significantly influence psychological distress, population based interventions are necessary to effect social and policy changes. Concurrently access to mental health support service for these populations is critical during and after the pandemic (51, 52). Happiness is certainly a variable that influences psychological distress in these communities. Future research will need to explore various solutions to mitigate the exacerbation of the mental health burden due to the COVID-19 pandemic amongst MSM and transgender communities This may include targeted online and telehealth services and/or 24/7 helplines which can be accessed regardless of restrictions. Further investigation around the potential protective factors of employment during COVID-19 on the impact of psychological distress and happiness is also needed to inform policy and practice.

## Conclusion

MSM and transgender individuals currently living in Bali, Indonesia are facing moderate to very high psychological distress and lack of happiness during the COVID-19 pandemic. Several factors contributed to the distress including being a student, reporting higher levels of stigma, had ever experienced discrimination, felt themselves better before the COVID-19 pandemic, and reporting less happy than average person. Factors contributed to reduced happiness including living with a partner and having moderate to very high psychological distress. These findings provide early evidence of the need for interventions aimed at improving general mental and sexual health amongst these communities during and after the pandemic. Stigma and discrimination are important areas of focus to reduce distress. Whilst these are not new issues for MSM and transgender communities, the COVID-19 pandemic is likely to exacerbate the impact. MSM and transgender people in Indonesia may become more hidden and find it difficult to access necessary sexual health services. Furthermore, restrictions may have further exacerbated the level of distress amongst those who are studying. Further research to explore the development of public health policy and the efficacy of interventions, particularly those that can be implemented through the NGO research partners, to support MSM and transgender people in Bali is required. This may include increased access to services including provision of online or “remote” services for MSM and transgender people. Broader governmental strategies to address employment during the pandemic should also be considered.

## Data Availability Statement

The supporting data of this study are available on request from the corresponding author. Access data set requests should be directed to Ni Wayan Septarini, septarini@unud.ac.id.

## Ethics Statement

The studies involving human participants were reviewed and approved by Human Ethics Committees, Faculty of Medicine, Udayana University/Sanglah Hospital, Bali, Indonesia (No: 2521/UN14.2.2.VII.14/LP/2019) and Curtin University, Western Australia (HRE 2019-0759). The patients/participants provided their written informed consent to participate in this study.

## Author Contributions

NS developed and drafting the proposal, data collection, analyses, and preparing/drafting the manuscript. JH, SB, and BM contributed equally to the acquisition and interpretation of data, revision on results, and critically reviewed the manuscript. All authors contributed to the article and approved the submitted version.

## Funding

This research project was funded through the Australia Awards Scholarship and administered through the doctoral program at the School of Public Health, Curtin University.

## Conflict of Interest

The authors declare that the research was conducted in the absence of any commercial or financial relationships that could be construed as a potential conflict of interest.

## Publisher's Note

All claims expressed in this article are solely those of the authors and do not necessarily represent those of their affiliated organizations, or those of the publisher, the editors and the reviewers. Any product that may be evaluated in this article, or claim that may be made by its manufacturer, is not guaranteed or endorsed by the publisher.
